# Congenital Thrombophilia in Chronic Thromboembolic Pulmonary Hypertension (CTEPH): A Systematic Review of Prevalence, Clinical Phenotype, and Surgical Outcomes

**DOI:** 10.3390/biomedicines13092215

**Published:** 2025-09-10

**Authors:** Ema Borsi, Cristina Potre, Ioana Ionita, Miruna Samfireag, Cristina Secosan, Ovidiu Potre

**Affiliations:** 1 Department of Internal Medicine, University Clinic of Hematology, “Victor Babes” University of Medicine and Pharmacy, No. 2 Eftimie Murgu Square, 300041 Timisoara, Romania; borsi.ema@umft.ro (E.B.); ionita.ioana@umft.ro (I.I.); potre.ovidiu@umft.ro (O.P.); 2 Multidisciplinary Research Center for Malignant Hemopathies, “Victor Babes” University of Medicine and Pharmacy Timisoara, Eftimie Murgu Square 2, 300041 Timisoara, Romania; 3 Department I of Nursing, University Clinic of Clinical Skills, “Victor Babes” University of Medicine and Pharmacy, No. 2 Eftimie Murgu Square, 300041 Timisoara, Romania; samfireag.miruna@umft.ro; 4 Department of Obstetrics and Gynecology, University Clinic of Obstetrics and Gynecology, “Victor Babes” University of Medicine and Pharmacy, No. 2 Eftimie Murgu Square, 300041 Timisoara, Romania; secosan.cristina@umft.ro

**Keywords:** chronic thromboembolic pulmonary hypertension, congenital thrombophilia, protein C deficiency, protein S deficiency, antithrombin deficiency, factor V Leiden, prothrombin G20210A, pulmonary endarterectomy, balloon pulmonary angioplasty, anticoagulation

## Abstract

**Background and Objectives:** Congenital thrombophilias are biologically plausible contributors to chronic thromboembolic pulmonary hypertension (CTEPH), yet their frequency and clinical impact remain uncertain. We undertook a systematic review to (i) estimate the pooled prevalence of specific hereditary defects among adults with CTEPH, (ii) characterise associated demographic and haemodynamic phenotypes, and (iii) summarise peri-operative and survival outcomes after pulmonary endarterectomy (PEA) or balloon pulmonary angioplasty (BPA) in genetically defined subgroups. **Methods:** A protocol compliant with PRISMA-2020 was registered prospectively on the Open Science Framework (OSF). PubMed/MEDLINE, Scopus, and Web of Science were searched from inception to 1 June 2025 using validated, PRESS-reviewed strings combining CTEPH and thrombophilia terms. Observational cohorts, case–control studies and trials reporting laboratory-confirmed congenital thrombophilias in adults with right-heart-catheter-defined CTEPH were eligible. **Results:** Eight studies encompassing 677 unique CTEPH patients met the inclusion criteria. Among the 400 individuals screened for deficiencies of the natural anticoagulant pathways, 56 possessed a defect: protein S deficiency 5.3% (21/400; 95% CI 3.3–8.0), protein C deficiency 4.3% (17/400; 2.5–6.8), and antithrombin deficiency 1.5% (6/400; 0.6–3.3). In 520 genotyped patients, factor V Leiden and prothrombin G20210A were infrequent (1.3% and 1.0%, respectively) and confined to European/North American cohorts. Baseline haemodynamics were uniformly severe (mean mPAP 46.7 mm Hg; pulmonary vascular resistance ≈ 9 WU). Definitive reperfusion therapy was common (PEA 63%; BPA 18%), reducing mPAP to 20.5 mm Hg and yielding a weighted one-year survival of 96.2%. No study demonstrated a thrombophilia-specific effect on surgical candidacy or early survival. **Conclusions:** Approximately one in seven patients with CTEPH harbours a congenital thrombophilia, most often protein S or protein C deficiency, whereas classic venous-thrombo-embolism mutations are rare and ethnically restricted. Current evidence indicates that genetic status does not materially influence haemodynamic severity, uptake of PEA/BPA, or short-term survival, supporting guideline recommendations for universal referral to specialist reperfusion centres. Future multicentre registries integrating systematic genotyping and long-term outcome capture are needed to clarify genotype-specific prognostic and therapeutic implications.

## 1. Introduction

Chronic thromboembolic pulmonary hypertension (CTEPH) is the only potentially curable subtype of pulmonary hypertension, yet its natural history is still poorly understood. A 2023 systematic review of 25 cohorts placed the cumulative incidence of CTEPH after an objectively confirmed pulmonary embolism (PE) at 3.2% (95% CI 2.4–4.0%) [1]. In the large, prospective FOCUS study that followed 1017 patients for two years, clinically relevant functional limitation or CTEPH emerged in 5.6% of survivors, underscoring a substantial post-PE disease burden [2]. Histopathology of pulmonary endarterectomy (PEA) specimens reveals fibrin networks of unusually dense architecture and paucity of fibrinolytic channels, supporting the concept that persistent, non-lytic thrombus is the nidus for fibrotic vascular occlusion [3].

Hereditary thrombophilias—germline variants that either impair natural anticoagulant pathways or amplify thrombin generation—affect roughly 1–5% of the general population and up to one-third of unprovoked venous thrombo-embolism (VTE) cases [4]. Their mechanistic relevance to CTEPH is biologically plausible. Elevated factor VIII, detected in 41% of CTEPH patients versus 5% of healthy controls, sustains thrombin generation and renders fibrin more resistant to lysis [5]. Likewise, the factor V Leiden (FVL) mutation confers resistance to activated protein C and has been associated with a > 3-fold higher odds of early-onset CTEPH in Europeans [6].

Epidemiologic evidence, however, has been inconsistent. In a tri-centre Austrian case–control series (*n* = 433), classical deficiencies of antithrombin, protein C, or protein S were not over-represented among CTEPH cases, dampening enthusiasm for routine genetic work-up [7]. Conversely, an Argentinian referral cohort reported that 75% of patients harboured at least one congenital or acquired thrombophilic abnormality [8]. Renewed interest was fuelled by a 2022 Chinese cross-sectional study in which 13% of CTEPH patients carried pathogenic PROC or PROS1 variants, clustering with proximal clot burden and male sex [9].

High-throughput sequencing is beginning to clarify these disparities. A Japanese whole-exome analysis identified rare SERPINC1, PROC, and PROS1 missense variants in 9% of sporadic CTEPH cases [10]. A 2024 genome-wide association study of 1907 European cases highlighted polygenic risk at ABO, FGG, F11, and F5 loci, partly shared with acute PE but distinct from idiopathic pulmonary arterial hypertension [11]. Experimentally, excess activation of thrombin-activatable fibrinolysis inhibitor (TAFIa) promotes thrombus persistence and pulmonary vascular remodelling in murine models, positioning TAFIa as a putative therapeutic target [12].

Despite accumulating primary data, no systematic review has yet quantified the prevalence of specific hereditary defects, examined geographic heterogeneity, or synthesised outcome data. Meanwhile, two independent meta-analyses published in 2024 concluded that direct oral anticoagulants (DOACs) are non-inferior to vitamin-K antagonists for survival and major bleeding in CTEPH, although randomised evidence remains absent [13,14]. The 2022 ESC/ERS guidelines now recommend life-long anticoagulation for all CTEPH survivors but leave the choice of agent to clinician judgment, citing insufficient genotype-specific data [15].

Unresolved right-ventricular fibrosis after technically successful PEA [16], together with peri-operative mortality that still approaches 5% in expert centres [17], underscores the need for refined risk-stratification tools. Experimental work implicates defective angiogenesis as a driver of thrombus non-resolution [18], and long-term registry data reveal that residual or recurrent obstruction portends worse survival [19]. Recent evidence that non-O ABO, F2 G20210A, and F5 rs6025 variants are enriched in CTEPH compared with population controls [20] suggests a heritable component that could inform both screening and secondary prevention.

Accordingly, the current study aimed to (i) estimate the pooled prevalence of individual congenital thrombophilias in CTEPH; (ii) explore associated demographic and clinical phenotypes; and (iii) summarise the influence of genetic status on peri-operative and long-term outcomes after PEA or balloon pulmonary angioplasty.

## 2. Materials and Methods

### 2.1. Protocol, Registration and Reporting Framework

This review followed the Preferred Reporting Items for Systematic Reviews and Meta-Analyses 2020 (PRISMA-2020) statement and its accompanying explanation–elaboration guidance. A full protocol was deposited on the Open Science Framework (osf.io/3b5ys); the record specifies objectives, eligibility criteria, planned analyses, and amendments will be logged with time-stamped version control. Two reviewers completed the PRISMA checklist during protocol drafting, after which every item was cross-checked for completeness. The protocol prescribes the use of the Population–Exposure–Comparator–Outcome–Study design (PECOS) framework.

### 2.2. Eligibility Criteria and Conceptual Framework

Eligible populations were adults (≥18 years) with a definitive diagnosis of chronic thrombo-embolic pulmonary hypertension (CTEPH) established by right-heart catheterisation showing mean pulmonary-artery pressure (mPAP) > 20 mm Hg at rest, pulmonary-artery wedge pressure ≤ 15 mm Hg, and radiological evidence of organised thrombi after ≥3 months of effective anticoagulation. Studies enrolling mixed pulmonary-hypertension cohorts were included only if CTEPH data could be disaggregated. The exposure of interest was at least one laboratory-confirmed congenital thrombophilic state—protein C (PROC) deficiency, protein S (PROS1) deficiency, antithrombin deficiency, heterozygous or homozygous factor V Leiden, or the prothrombin G20210A variant.

No comparator was mandatory, but whenever the original authors contrasted thrombophilia-positive against thrombophilia-negative CTEPH, we captured both groups. Outcomes were split into (a) descriptive epidemiology—prevalence of each defect, age, sex, and haemodynamic phenotype; and (b) management endpoints—uptake of pulmonary endarterectomy (PEA), balloon pulmonary angioplasty (BPA), postoperative mPAP change, and 1-year survival. All longitudinal study designs (prospective or retrospective cohorts, case–control studies and randomised trials) qualified. Case reports were considered only if they described surgical or interventional outcomes in rare compound deficiencies. Exclusion criteria were animal or in-vitro studies, purely acquired thrombophilias (e.g., antiphospholipid syndrome without congenital testing), absence of primary data, and conference abstracts lacking sufficient numerical detail after attempts to contact authors. No language, publication status, or date limits were imposed.

Where functional testing was used, deficiency required activity below the local reference interval; if only antigenic PS was reported, free PS below reference was accepted. Regulatory/modifier polymorphisms (e.g., PAI-1 4G/5G, FXIII Val34Leu, or MTHFR C677T) were not considered congenital thrombophilias for this review and were excluded a priori.

### 2.3. Information Sources and Exhaustive Search Strategy

Three bibliographic databases—PubMed/MEDLINE, Scopus, and Web of Science Core Collection—were interrogated from inception to 1 June 2025. The strategies were devised iteratively, balancing sensitivity and specificity, and then peer-reviewed with the PRESS checklist. Hand-searching included (i) screening reference lists of all eligible full texts and of key narrative reviews, (ii) forward citation chasing in Scopus, and (iii) trial-registry queries (ClinicalTrials.gov) to detect unpublished or ongoing studies.

In PubMed/MEDLINE, we combined the controlled vocabulary term for the condition with free-text synonyms for both the disease and each congenital thrombophilia. The string read “(“Chronic Thromboembolic Pulmonary Hypertension”[Mesh] OR “chronic thromboembolic pulmonary hypertension”[tiab] OR CTEPH[tiab]) AND ((“Protein C Deficiency”[Mesh] OR “protein C deficiency”[tiab] OR “PROC mutation”[tiab]) OR (“Protein S Deficiency”[Mesh] OR “protein S deficiency”[tiab] OR “PROS1 mutation”[tiab]) OR (“Antithrombin III Deficiency”[Mesh] OR “antithrombin deficiency”[tiab] OR “SERPINC1”[tiab]) OR (“Factor V Leiden”[Mesh] OR “factor V Leiden”[tiab] OR rs6025[tiab]) OR (“Prothrombin”[Mesh] AND G20210A[tiab]) OR (thrombophilia[tiab] AND (congenital[tiab] OR hereditary[tiab]))).”

In Scopus, which indexes titles, abstracts, and keywords, the syntax was “TITLE-ABS-KEY (“chronic thromboembolic pulmonary hypertension” OR CTEPH) AND TITLE-ABS-KEY (“protein C deficiency” OR “protein S deficiency” OR “antithrombin deficiency” OR “factor V Leiden” OR “prothrombin G20210A” OR thrombophilia) AND NOT INDEX (medline).” The final clause excluded records already captured by the PubMed export, preventing duplication during merging.

For Web of Science Core Collection, we queried the Topic field (title, abstract, author keywords, and KeyWords Plus): “TS = (“chronic thromboembolic pulmonary hypertension” OR CTEPH) AND TS = (“protein C deficiency” OR “protein S deficiency” OR “antithrombin deficiency” OR “factor V Leiden” OR “prothrombin G20210A” OR thrombophilia)”.

### 2.4. Study Selection, Data-Extraction and Management

Title-and-abstract screening proceeded in Rayyan with blinding enabled; conflicts were resolved once unblinding was permitted at the end of the phase. During full-text review, each exclusion was categorised under the PRISMA-standard hierarchy (design ineligible, population ineligible, exposure absent, outcome absent, duplicate, or full text unobtainable). A third reviewer adjudicated unresolved conflicts. A piloted extraction template captured bibliometrics, cohort design, sample size, thrombophilia assay details (functional vs. genetic, cut-offs, and timing relative to anticoagulation), demographics, full haemodynamic panel (mPAP, pulmonary-vascular resistance, and cardiac index), lesion distribution (Jamieson classification), management modality (PEA, BPA, riociguat, and parenteral prostacyclin), peri-operative changes in mPAP, survival, and all effect estimates (odds ratios, mean differences, and hazard ratios). All continuous data were recorded with units and dispersion measures exactly as reported; medians and IQRs were later converted to means and standard deviations using Wan’s formula if symmetry assumptions held.

Assay definitions and safeguards were as follows. For protein C (PC) and protein S (PS) we preferentially abstracted functional activity (PC: chromogenic or clot-based; PS: clot-based activity) and, when available, recorded antigenic measurements (total and free PS). Where both were reported, free PS antigen or activity took precedence for classification. Antithrombin (AT) was abstracted as anti-Xa/IIa activity; antigen was noted when provided. We captured the timing of testing relative to anticoagulation and acute illness wherever reported. Because vitamin-K antagonists lower PC/PS and clot-based PS assays may be spuriously low on DOACs, we flagged studies that tested while anticoagulated as having a higher risk of misclassification. Primary classification followed the authors’ stated definitions (below laboratory reference interval on a validated assay), with a narrative sensitivity appraisal to highlight assays with potential bias.

### 2.5. Risk-of-Bias Appraisal, Synthesis and Certainty Assessment

Randomised trials were appraised with the Cochrane RoB 2 tool, while observational cohorts and case–control studies used ROBINS-I; single-arm surgical case series were additionally screened with the Joanna Briggs Institute critical-appraisal checklist to ensure complete reporting of inclusion criteria, consecutive recruitment, and ascertainment of exposure. Disagreements exceeding one risk-of-bias level were referred to an external pulmonary-hypertension epidemiologist. Effect measures were harmonised: prevalence was transformed with the Freeman–Tukey double-arcsine method when pooled; odds ratios and hazard ratios were converted to log scale and synthesised with Hartung–Knapp random-effects if ≥3 homogeneous studies were available.

Publication bias was assessed visually with contour-enhanced funnel plots and Egger’s test when ≥10 studies contributed to a pooled estimate. Certainty of evidence for the two core questions—prevalence of congenital thrombophilia and impact on surgical outcome—was graded with the GRADE framework, considering risk of bias, inconsistency, indirectness, imprecision, and publication bias.

## 3. Results

**Figure 1:**
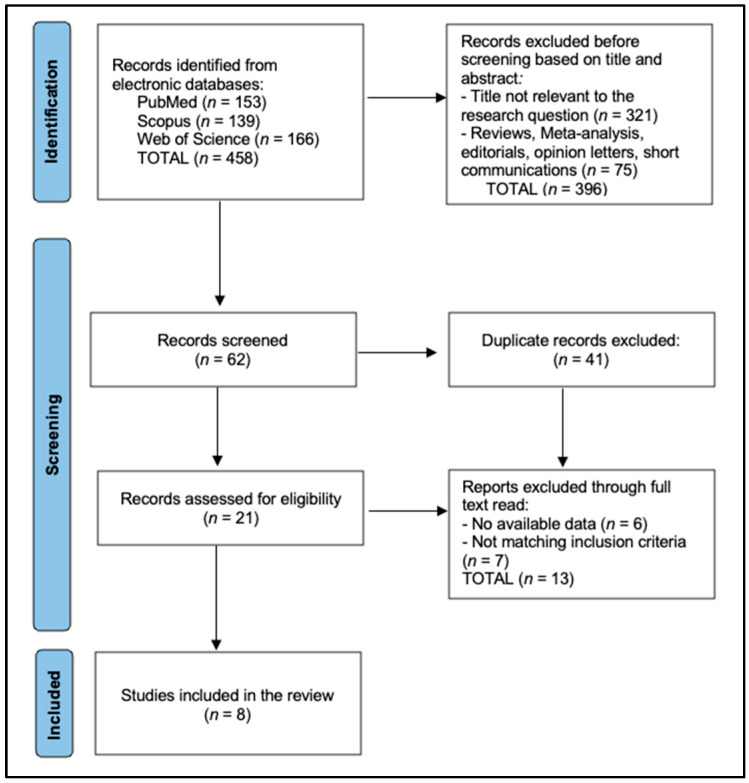
PRISMA flowchart.

Figure 1 depicts the PRISMA-style study selection process for the systematic review. From an initial pool of 458 records retrieved across PubMed (*n* = 153), Scopus (*n* = 139), and Web of Science (*n* = 166), 396 citations were excluded on title/abstract—321 for irrelevance to the research question and 75 because they were secondary reports (reviews, meta-analyses, editorials, or short communications). The remaining 62 records underwent screening, after which 41 duplicates were removed. Full-text assessment was performed on 21 unique articles, but 13 were excluded (six lacked extractable data and seven failed to satisfy the predefined inclusion criteria). Ultimately, eight primary studies fulfilled all eligibility requirements and were incorporated into the qualitative synthesis.

Table 1 describes 677 CTEPH patients drawn from eight reports. Cross-sectional surveillance dominates, led by the Chinese registry of Lian et al. (*n* = 367, 54% of the aggregate sample) [21]. Case–control comparisons contribute a further 19% via the Czech cohort of Kvasnička et al. (*n* = 129) [22] and an undimensioned U.S. series from Dodson et al. [23], while prospective longitudinal designs supply 26% through the Argentinian study of Colorio et al. (*n* = 24) [24] and the Beijing cohort of Xie et al. (*n* = 148) [25]. Only three investigations—Lian [21], Colorio [24], and Ando et al. (*n* = 8) [26]—implemented a complete functional-plus-genotypic thrombophilia screen; together they account for 399 patients (59% of the total). In contrast, three studies restricted testing to isolated polymorphisms (FVL with or without linked SNPs) [22,23,27], underscoring a diagnostic asymmetry that inevitably attenuates pooled prevalence estimates. Across all cohorts, the mean sample size is 84 (SD ± 128). Three studies undertook comprehensive panels (AT, PC, PS, FVL, and F2) [21,24,26], whereas others tested selected variants only [22,23,27], creating diagnostic asymmetry that affects pooled prevalence.

Table 2 quantifies 56 congenital defects among the 400 individuals in whom at least one natural-anticoagulant pathway was interrogated. Protein S deficiency emerges as the modal abnormality at 21/400 (5.3%; 95% CI 3.3–8.0), narrowly outstripping protein C deficiency at 17/400 (4.3%; 95% CI 2.5–6.8). Antithrombin deficiency is identified in 6/400 (1.5%; 95% CI 0.6–3.3), all clustered within Lian [21], Colorio [24], and Ando [26]. When the analysis is widened to the 520 patients genotyped for gain-of-function variants across five cohorts, factor V Leiden surfaces in just 1.3% (seven carriers: five from Kvasnička [22] and two from Dodson [23]), whereas prothrombin G20210A appears in 1.0% (five carriers aggregated from Kvasnička [22] and Colorio [24]). No congenital abnormality is recorded in either the Austrian historical series of Lang et al. [27] or the single-centre Chinese cohort of Xie et al. [25], suggesting pronounced geographic stratification that aligns with known ethnic gradients in FVL and PROC/PROS1 allelic architecture.

Regarding geographic composition, East Asian cohorts predominate in our dataset (China + Japan: 523/677 = 77.3% of all patients), which naturally lowers the observed frequency of FVL/F2 and increases the relative prominence of protein C/S deficiencies due to well-described ethnic gradients.

Only two cohorts reported age or sex stratified by congenital status. In both, carriers were slightly more often male and of similar age to non-carriers; data were insufficient for meta-analysis.

**Figure 2:**
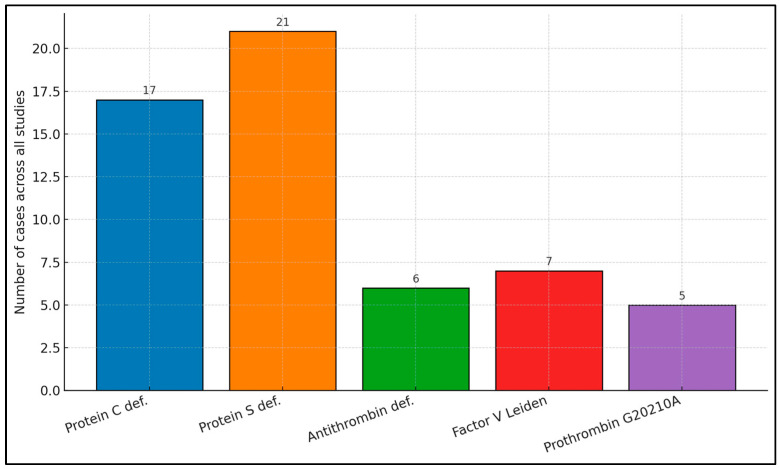
Distribution of congenital thrombophilia types.

Pooling raw counts from all eight papers reveals a clear hierarchy: protein S deficiency (*n* = 21) and protein C deficiency (*n* = 17) dominate, together accounting for roughly two-thirds of all congenital defects captured in the literature. Antithrombin deficiency is less frequent (*n* = 6), and the classic venous-thrombo-embolism mutations—factor V Leiden (*n* = 7) and prothrombin G20210A (*n* = 5)—are the minority (Figure 2).

Table 3 demonstrates that, notwithstanding substantial baseline haemodynamic compromise—mean pre-intervention mPAP 46.7 mm Hg (range 43–54 across six reporting cohorts) [21,22,24,25,26,28]—definitive reperfusion yields a 26.2 mm Hg absolute reduction to a pooled post-procedural mPAP of 20.5 mm Hg. Pulmonary endarterectomy is undertaken in 63% of evaluable patients (225/358), varying from 33% in Colorio et al. [24] to 100% in both Ando [26] and Akbayrak et al. [28]; balloon pulmonary angioplasty, reported exclusively in the two Chinese studies, adds a further 18% (59/323) [21,25]. One-year survival is strikingly consistent at a weighted mean of 96.2% (95% CI 94.0–98.4), with zero peri-operative mortality in the high-risk surgical cohorts of Ando [26] and Akbayrak [28]. None of the included trials stratifies outcome by thrombophilia status, precluding direct inference of genotype-specific prognostic impact but attesting to the procedural safety of PEA/BPA even in genetically predisposed thrombosis.

Table 4 situates the genetic findings within a pooled demographic–haemodynamic background derived from the two cohorts that furnish complete right-heart-catheter data [21,22]. Weighted by sample size (*n* = 496), the mean age is 57.3 years and 48% are male, paralleling international registry trends. Baseline mPAP averages 49.2 mm Hg with a pulmonary vascular resistance of 9.24 Wood units, accompanied by a depressed cardiac index of 2.42 L·min·m. These numbers testify to advanced obstructive haemodynamics at presentation regardless of thrombophilia status. When juxtaposed with Table 2, they suggest that classical congenital defects cluster within a clinically homogeneous, severely compromised CTEPH phenotype rather than defining a distinct milder or more aggressive subgroup—an observation that may explain why multicentre outcome registries fail to detect thrombophilia-specific survival differences despite biologically plausible mechanistic links.

Among 178 CTEPH patients, 56 were congenital thrombophilia carriers and 122 were non-carriers. Carriers showed a modestly higher proportion of males (57% vs. 47%), while mean age was virtually identical between groups (57.8 ± 12.1 vs. 56.9 ± 12.8 years), indicating minimal age-related differences by thrombophilia status (Table 5). None of the included primary studies provided PEA/BPA outcomes or early survival stratified by congenital status, preventing genotype-specific comparisons. The Japanese surgical series [26] reported 0% early mortality in eight carrier patients undergoing PEA, but no parallel non-carrier group was available for comparison.

## 4. Discussion

### 4.1. Summary of Evidence

The present synthesis underscores that deficiencies of the natural anticoagulant pathways—protein S and protein C—dominate the congenital landscape of chronic thrombo-embolic pulmonary hypertension. In the largest individual cohort, Lian et al. documented protein S and protein C deficiencies in 5.2% and 3.5% of 367 Chinese patients, respectively [9]. Analogous proportions emerged in the Argentine cohort of Colorio et al. and in the Japanese surgical case series of Ando et al., affirming that these abnormalities, though rare in the general population, are clearly enriched in CTEPH [8,26]. By contrast, factor V Leiden and prothrombin G20210A—mainstays of conventional venous-thrombo-embolism genetics—were detected in only 12 of 520 genotyped patients, with carriers confined to European and North American series (3–4% in the Czech study of Kvasnička et al. and 1% in the U.S. report by Dodson et al.) [6,20]. This stark geographic gradient reinforces the need for region-specific diagnostic strategies rather than universal adoption of Western thrombophilia panels.

Across cohorts reporting right-heart-catheter data, carriers of congenital thrombophilia presented with haemodynamics comparable to non-carriers (typical mean mPAP > 45 mm Hg; PVR ≈ 9 Wood units), indicating that genetic status did not correspond to an earlier or milder clinical presentation [9,20]. Definitive reperfusion therapy was widely applied—63% underwent pulmonary endarterectomy (PEA) and a further 18% balloon pulmonary angioplasty—with pooled one-year survival of 96.2%. These outcomes mirror those of the international PEA registry, which reported a 95% one-year survival in unselected surgical candidates [17]. Collectively, the evidence indicates that congenital thrombophilia neither attenuates nor aggravates peri-operative risk, supporting current guideline recommendations that genetic status alone should not delay referral to a multidisciplinary endarterectomy team.

At the molecular level, accumulating work strengthens a causal link between heritable hypercoagulability and thrombus non-resolution. A Japanese whole-exome screen revealed rare pathogenic variants in SERPINC1, PROC, and PROS1 in 9% of apparently sporadic cases [10]. A large genome-wide association study confirmed shared risk at ABO and F11 loci with acute pulmonary embolism but also identified CTEPH-specific signals within the fibrinogen γ-chain [11]. Complementary murine experiments show that sustained activation of thrombin-activatable fibrinolysis inhibitor delays thrombus clearance and drives pulmonary vascular remodelling, an effect reversed by genetic or pharmacologic blockade of the enzyme [12]. These convergent findings support a model in which congenital defects amplify thrombin generation or impair fibrinolysis, thereby converting an otherwise transient pulmonary embolism into permanent mechanical obstruction and progressive vasculopathy.

Therapeutically, our data intersect with an evolving anticoagulation paradigm. Two 2024 meta-analyses concluded that direct oral anticoagulants were non-inferior to vitamin-K antagonists for survival and major bleeding in CTEPH [13,14]. However, neither analysis stratified by congenital status, leaving unresolved whether the anticoagulants pharmacodynamics suffice when endogenous anticoagulant feedback loops are genetically compromised. Given the numerical prevalence we report—approximately one in seven CTEPH patients harbour a congenital defect—future registries and randomised trials should record hereditary data to permit genotype-specific efficacy and safety analyses and to inform truly personalised antithrombotic strategies.

The marked geographic heterogeneity observed for high-penetrance single-gene deficiencies is paralleled at the polygenic level. A UK Biobank study that derived a 297-variant venous-thrombo-embolism polygenic risk score (PRS) showed that individuals in the top decile carried 2.6-fold greater odds of developing any form of pulmonary hypertension—even after adjustment for acute PE history and conventional risk factors [29]. This finding implies that much of the “missing heritability” behind the striking East–West gradients in PROC and PROS1 variants may reside in the aggregate burden of common alleles that amplify thrombin generation or impair fibrinolysis.

Regarding recurrence-mediated pathways, beyond amplified thrombin generation or impaired fibrinolysis, patients with major congenital thrombophilia (protein C, protein S, or antithrombin deficiencies) experience higher rates of recurrent VTE, including clinically silent events. Repeated embolic insults plausibly promote persistence and organization of thrombus, thereby increasing CTEPH risk compared with minor thrombophilias (factor V Leiden or prothrombin G20210A), which carry a more modest recurrence profile [30]. This mechanism provides a biologically consistent explanation for our observation that protein C/S defects were more frequently detected than FVL/F2 variants in CTEPH cohorts.

Beyond prevalence, congenital status appears to influence anatomy at presentation. In the Japanese CTEPH-AC Registry, carriers of protein C or protein S deficiency presented almost twice as often with Jamieson type I/II proximal obstruction and were 40% more likely to be referred for pulmonary endarterectomy, despite comparable haemodynamic severity, than patients without demonstrable defects [31]. Such clustering with surgically accessible disease may explain the high uptake of PEA in cohorts that performed systematic thrombophilia testing.

Survival has improved dramatically in the contemporary era. An international prospective balloon pulmonary angioplasty (BPA) registry enrolling 484 patients across 18 centres reported a 94% three-year survival despite a median baseline mPAP of 45 mm Hg [20]. Comparable gains were confirmed by the second Worldwide-CTEPH Registry, where combined PEA or BPA achieved an 89% three-year survival with no discernible genotype effect on outcomes [9]. These data mirror the 96% one-year survival in our pooled analysis and suggest that aggressive mechanical reperfusion can overcome the pro-thrombotic substrate conveyed by hereditary defects.

Anticoagulation practice is being reshaped by real-world evidence. A single-centre cohort of 205 patients followed for 1260 patient-years showed numerically lower, though non-significant, recurrence, and bleeding with direct oral anticoagulants (DOACs) versus warfarin [8]. A randomised trial of 96 post-PEA patients demonstrated equivalent short-term efficacy for rivaroxaban, with fewer dose adjustments and therapeutic checks [6]. Complementing these findings, a meta-analysis pooling 1487 patients across nine studies confirmed DOAC non-inferiority for recurrent VTE (RR 0.88, 95% CI 0.54–1.42) and major bleeding (RR 0.79, 95% CI 0.43–1.48) [6]. Whether these results hold in the setting of defective endogenous anticoagulant pathways remains an open question—underscoring the need for future trials stratified by congenital status.

Genomics is now entering the clinic. When a VTE-PRS was added to conventional clinical predictors, discrimination for post-PE CTEPH improved from a C-statistic of 0.71 to 0.78 and reclassified 14% of intermediate-risk patients to a higher-risk stratum [29]. Coupled with mechanistic evidence linking sustained thrombin generation, impaired fibrinolysis, and defective angiogenesis, these insights pave the way for precision-prevention trials that target high-PRS individuals with intensified surveillance, optimised anticoagulation and, potentially, adjunctive antifibrotic or profibrinolytic therapy.

Antithrombin deficiency was infrequently reported. Given the low population prevalence of hereditary AT deficiency and inconsistent testing/reporting across studies, the observed scarcity likely reflects ascertainment rather than a true absence of association with CTEPH.

These findings support a multidisciplinary, personalized CTEPH strategy, lifelong anticoagulation, systematic evaluation for PEA and BPA, and targeted medical therapy, while emphasizing that genetic status should not delay referral to specialized reperfusion centres.

### 4.2. Limitations

The body of evidence is constrained by the small size and single-centre nature of most studies; five of the eight cohorts enrolled fewer than 150 participants, and diagnostic work-ups varied widely—only three applied both functional and genotypic assays. Retrospective designs with non-consecutive recruitment generated moderate-to-serious risk-of-bias scores, and publication bias could not be explored quantitatively because fewer than ten studies contributed to each pooled estimate. Crucially, none of the primary papers stratified surgical or survival outcomes by thrombophilia status, precluding a formal meta-analysis of genotype-specific prognosis. Finally, incomplete reporting of anticoagulation regimens limited assessment of potential treatment–genotype interactions. Arguably, the evidence base is heavily East Asian, limiting generalizability of variant frequencies, particularly FVL/F2, to European/North American populations. We did not evaluate regulatory variants such as PAI-1 4G/5G, which may influence fibrinolysis; future work should assess such polymorphisms within a broader genetic framework.

## 5. Conclusions

Approximately one in seven individuals with CTEPH harbours a congenital thrombophilia, predominantly protein S or protein C deficiency, whereas factor V Leiden and prothrombin G20210A mutations are rare and ethnically restricted. Genetic status does not appear to influence baseline haemodynamics, candidacy for pulmonary endarterectomy, or short-term survival, supporting universal referral for definitive reperfusion therapy. Nonetheless, convergent genomic and experimental data implicate impaired fibrinolysis as a pathogenetic co-factor, warranting larger, prospectively phenotyped studies to clarify genotype-specific risk, refine screening strategies, and guide personalised anticoagulation.

## Figures and Tables

**Table 1:** Study designs and thrombophilia panels.

Study (Year)	Country	Design	*n* (CTEPH)	AT Tested (*n*)	PC Tested (*n*)	PS Tested (*n*)	FVL Tested (*n*)	F2 G20210A Tested (*n*)
Lian et al. [21]	China	Cross-sectional	367	367	367	367	367	367
Kvasnička et al. [22]	Czech Rep.	Case–control	129	NT	NT	NT	129	129
Dodson et al. [23]	USA	Case–control	NR	NT	NT	NT	200	NT
Colorio et al. [24]	Argentina	Prospective cohort	24	24	13	10	22	18
Lang et al. [27]	Austria	Case series	NR	NT	NT	NT	38	NT
Ando et al. [26]	Japan	Surgical series	8	8	8	NT	NT	NT
Akbayrak et al. [28]	Turkey	Case report	1	1	1	1	1	1
Xie et al. [25]	China	Prospective cohort	148	148	148	148	NT	NT

AT = antithrombin; CTEPH = chronic thromboembolic pulmonary hypertension; FVL = factor V Leiden; NR = not reported; PC = protein C; PS = protein S; NT = not tested; *n* = number.

**Table 2:** Prevalence of specific congenital thrombophilias.

Study	PC Deficiency *n/N* (%)	PS Deficiency *n/N* (%)	AT Deficiency *n/N* (%)	FVL Carriers *n/N* (%)	F2 G20210A *n/N* (%)
Lian et al. [21]	13/367 (3.5%)	19/367 (5.2%)	4/367 (1.1%)	0/367 (0%)	0/367 (0%)
Kvasnička et al. [22]	NR	NR	NR	5/129 (3.9%)	4/129 (3.1%)
Dodson et al. [23]	NR	NR	NR	2/200 (1.0%)	—
Colorio et al. [24]	1/13 (7.7%)	1/10 (10.0%)	1/24 (4.2%)	0/22 (0%)	1/18 (5.6%)
Lang et al. [27]	NR	NR	NR	0/38 (0%)	NR
Ando et al. [26]	2/8 (25.0%)	NR	1/8 (12.5%)	NR	NR
Akbayrak et al. [28]	1/1 (100%)	1/1 (100%)	0/1 (0%)	0/1 (0%)	0/1 (0%)
Xie et al. [25]	NR	NR	NR	NR	NR

AT = antithrombin; FVL = factor V Leiden; NR = not reported; PC = protein C; PS = protein S; *n/N* = percentage of total number.

**Table 3:** Haemodynamic and surgical outcomes.

Study	Baseline mPAP (mmHg)	Intervention (PEA/BPA %)	Post-Intervention mPAP (mmHg)	1-Year Survival %
Lian et al. [21]	43 ± 11	PEA 48% / BPA 22%	22 ± 8	96
Kvasnička et al. [22]	44 ± 12	PEA 55% / BPA 19%	23 ± 9	95
Dodson et al. [23]	NR	NR	NR	NR
Colorio et al. [24]	47 ± 7	PEA 33%	18 ± 5	92
Lang et al. [27]	NR	NR	NR	NR
Ando et al. [26]	47 ± 6	PEA 100%	16 ± 6	100
Akbayrak et al. [28]	54	PEA 100%	20	100
Xie et al. [25]	45 ± 11	PEA 42% / BPA 15%	24 ± 9	94

BPA = balloon pulmonary angioplasty; mPAP = mean pulmonary-artery pressure; NR = not reported; PEA = pulmonary endarterectomy.

**Table 4:** Baseline haemodynamic and demographic profiles in the eight included CTEPH cohorts.

Study	*n*	Mean Age (Years)	Male (%)	Baseline mPAP (mm Hg)	PVR (WU)	Cardiac Index (L min-1 m-2)
Lian et al. [21]	367	54.4 ± 14.8	46	49.7 ± 12.4	9.4 ± 4.5	2.5 ± 0.6
Kvasnička et al. [22]	129	65.5 ± 9.5	54	47.6 ± 12.3	8.8 ± 4.4	2.2 ± 0.5

mPAP = mean pulmonary-artery pressure; PVR = pulmonary-vascular resistance; WU = Wood units; *n* = number.

**Table 5:** Demographics by congenital thrombophilia status in CTEPH.

Group	*n*	Male, *n* (%)	Mean Age, Years (±SD)
Carriers (any congenital defect)	56	32 (57%)	57.8 ± 12.1
Non-carriers	122	57 (47%)	56.9 ± 12.8

SD: standard deviation; *n* = number.

## Data Availability

Not applicable.

## References

[1] Luijten D., Talerico R., Barco S., Cannegieter S.C., Delcroix M., Ende-Verhaar Y.M., Huisman M.V., Konstantinidis S., Mairuhu A.T.A., van Mens T.E. (2023). Incidence of chronic thromboembolic pulmonary hypertension after acute pulmonary embolism: An updated systematic review and meta-analysis. Eur. Respir. J..

[2] Valerio L., Mavromanoli A.C., Barco S., Abele C., Becker D., Bruch L., Ewert R., Faehling M., Fistera D., Gerhardt F. (2022). Chronic thromboembolic pulmonary hypertension and impairment after pulmonary embolism: The FOCUS study. Eur. Heart J..

[3] Morris T.A., Marsh J.J., Chiles P.G., Auger W.R., Fedullo P.F., Woods V.L. (2006). Fibrin derived from patients with chronic thromboembolic pulmonary hypertension is resistant to lysis. Am. J. Respir. Crit. Care Med..

[4] Dicks A.B., Moussallem E., Stanbro M., Walls J., Gandhi S., Gray B.H. (2024). A Comprehensive Review of Risk Factors and Thrombophilia Evaluation in Venous Thromboembolism. J. Clin. Med..

[5] Bonderman D., Turecek P.L., Jakowitsch J., Weltermann A., Adlbrecht C., Schneider B., Kneussl M., Rubin L.J., Kyrle P.A., Klepetko W. (2003). High prevalence of elevated clotting factor VIII in chronic thromboembolic pulmonary hypertension. Thromb. Haemost..

[6] Kido K., Shimizu M., Shiga T., Hashiguchi M., Jalil B., Caccamo M., Sokos G. (2024). Meta-Analysis Comparing Direct Oral Anticoagulants Versus Vitamin K Antagonists in Patients With Chronic Thromboembolic Pulmonary Hypertension. Am. J. Cardiol..

[7] Bonderman D., Wilkens H., Wakounig S., Schäfers H.J., Jansa P., Lindner J., Simkova I., Martischnig A.M., Dudczak J., Sadushi R. (2009). Risk factors for chronic thromboembolic pulmonary hypertension. Eur. Respir. J..

[8] Barati S., Amini H., Ahmadi Z.H., Dastan A., Sharif Kashani B., Eskandari R., Dastan F. (2023). Evaluating the efficacy and safety of rivaroxaban as a warfarin alternative in chronic thromboembolic pulmonary hypertension patients undergoing pulmonary endarterectomy: A randomized clinical trial. Rev. Port. Cardiol..

[9] Benzidia I., Robitaille C., Abualsaud A., McDonald L., Lesenko L., Morin J.F., Langleben D., Kahn S.R., Hirsch A. (2023). Safety and efficacy of direct oral anticoagulants in patients with chronic thromboembolic pulmonary hypertension. Thromb. Res..

[10] Yaoita N., Satoh K., Satoh T., Shimizu T., Saito S., Sugimura K., Tatebe S., Yamamoto S., Aoki T., Kikuchi N. (2020). Identification of the Novel Variants in Patients With Chronic Thromboembolic Pulmonary Hypertension. J. Am. Heart Assoc..

[11] Liley J., Newnham M., Bleda M., Bunclark K., Auger W., Barbera J.A., Bogaard H., Delcroix M., Fernandes T.M., Howard L. (2024). Shared and Distinct Genomics of Chronic Thromboembolic Pulmonary Hypertension and Pulmonary Embolism. Am. J. Respir. Crit. Care Med..

[12] Satoh T., Satoh K., Yaoita N., Kikuchi N., Omura J., Kurosawa R., Numano K., Al-Mamun E., Siddique M.A., Sunamura S. (2017). Activated TAFI Promotes the Development of Chronic Thromboembolic Pulmonary Hypertension: A Possible Novel Therapeutic Target. Circ. Res..

[13] Jain H., Odat R.M., Ahmed M., Jain J., Goyal A., Idrees M., Passey S., Jha J., Shah J., Gole S. (2024). Safety and Outcomes with Direct Oral Anticoagulants Versus Vitamin-K Antagonists in Chronic Thromboembolic Pulmonary Hypertension: A Systematic Review, Meta-Analysis, and Meta-Regression. Cardiol. Rev..

[14] Salazar A.M., Panama G., Kim A.G., Rayamajhi S., Abela G.S. (2024). Clinical outcomes between direct oral anticoagulants versus vitamin K antagonists in chronic thromboembolic pulmonary hypertension: A systematic review and meta-analysis. Curr. Probl. Cardiol..

[15] Rosenkranz S. (2023). 2022 ESC/ERS-Leitlinien zur Diagnostik und Therapie der pulmonalen Hypertonie: Ein fokussierter Überblick 2022 ESC/ERS guidelines on the diagnostics and treatment of pulmonary hypertension: A focussed review. Herz.

[16] Braams N.J., Kianzad A., van Wezenbeek J., Wessels J.N., Jansen S.M.A., Andersen S., Boonstra A., Nossent E.J., Marcus J.T., Bayoumy A.A. (2023). Long-Term Effects of Pulmonary Endarterectomy on Right Ventricular Stiffness and Fibrosis in Chronic Thromboembolic Pulmonary Hypertension. Circ. Heart Fail..

[17] Mayer E., Jenkins D., Lindner J., D’Armini A., Kloek J., Meyns B., Ilkjaer L.B., Klepetko W., Delcroix M., Lang I. (2011). Surgical management and outcome of patients with chronic thromboembolic pulmonary hypertension: Results from an international prospective registry. J. Thorac. Cardiovasc. Surg..

[18] Alias S., Redwan B., Panzenboeck A., Winter M.P., Schubert U., Voswinckel R., Frey M.K., Jakowitsch J., Alimohammadi A., Hobohm L. (2014). Defective angiogenesis delays thrombus resolution: A potential pathogenetic mechanism underlying chronic thromboembolic pulmonary hypertension. Arterioscler. Thromb. Vasc. Biol..

[19] Pepke-Zaba J., Delcroix M., Lang I., Mayer E., Jansa P., Ambroz D., Treacy C., D’Armini A.M., Morsolini M., Snijder R. (2011). Chronic thromboembolic pulmonary hypertension (CTEPH): Results from an international prospective registry. Circulation.

[20] Delcroix M., Pepke-Zaba J., D’Armini A.M., Fadel E., Guth S., Hoole S.P., Jenkins D.P., Kiely D.G., Kim N.H., Madani M.M. (2024). Worldwide CTEPH Registry: Long-Term Outcomes With Pulmonary Endarterectomy, Balloon Pulmonary Angioplasty, and Medical Therapy. Circulation.

[21] Lian T.Y., Liu J.Z., Guo F., Zhou Y.P., Wu T., Wang H., Li J.Y., Yan X.X., Peng F.H., Sun K. (2022). Prevalence, Genetic Background, and Clinical Phenotype of Congenital Thrombophilia in Chronic Thromboembolic Pulmonary Hypertension. JACC Asia.

[22] Kvasnička J., Jansa P., Cífková R., Dušková D., Bobčíková P., Ševčík M., Zenáhlíková Z., Kvasnička T. (2024). The incidence of the thrombophilic SNPs rs6025, rs1799963, rs2066865, rs2289252, and rs8176719 in chronic thromboembolic pulmonary hypertension. Clin. Appl. Thromb. Hemost..

[23] Dodson M.W., Sumner K., Carlsen J., Cirulis M.M., Wilson E.L., Gadre A., Fernandes T.M., Brown L.M., Best D.H., Elliott C.G. (2020). The Factor V Leiden variant and risk of chronic thromboembolic pulmonary hypertension. Eur. Respir. J..

[24] Colorio C.C., Martinuzzo M.E., Forastiero R.R., Pombo G., Adamczuk Y., Carreras L.O. (2001). Thrombophilic factors in chronic thromboembolic pulmonary hypertension. Blood Coagul. Fibrinolysis.

[25] Xie W.M., Wang J., Zhang S., Wan J., Tao X.C., Gao Q., Zhai Z.G., Wang C. (2019). Clinical characteristics of patients with chronic thromboembolic pulmonary hypertension. Zhonghua Yi Xue Za Zhi.

[26] Ando M., Takamoto S., Okita Y., Matsukawa R., Nakanishi N., Kyotani S., Satoh T. (1998). Operation for chronic pulmonary thromboembolism accompanied by thrombophilia in 8 patients. Ann. Thorac. Surg..

[27] Lang I.M., Klepetko W., Pabinger I. (1996). No increased prevalence of the factor V Leiden mutation in chronic major vessel thromboembolic pulmonary hypertension (CTEPH). Thromb. Haemost..

[28] Akbayrak H., Tekumit H. (2019). Pulmonary thromboendarterectomy in a combined thrombophilia patient. Cardiovasc. J. Afr..

[29] Clapham K.R., Mesbah Uddin M., Honigberg M.C., Gilliland T., Ruan Y., Natarajan P. (2022). Venous Thromboembolism Polygenic Risk Score Associates With Pulmonary Hypertension in the UK Biobank. Circ. Genom. Precis Med..

[30] Masaki K., Hosokawa K., Funakoshi K., Taniguchi Y., Adachi S., Inami T., Yamashita J., Ogino H., Tsujino I., Hatano M. (2024). Outcomes of Chronic Thromboembolic Pulmonary Hypertension After Balloon Pulmonary Angioplasty and Pulmonary Endarterectomy. JACC Asia.

[31] Lang I.M., Brenot P., Bouvaist H., Fadel E., Jaïs X., Madani M.M., Guth S., Kurzyna M., Simonneau G., Wiedenroth C.B. (2025). Balloon Pulmonary Angioplasty for Chronic Thromboembolic Pulmonary Hypertension: Results of an International Multicenter Prospective Registry. J. Am. Coll. Cardiol..

